# At home and at risk: The experiences of Irish adults living with obesity during the COVID-19 pandemic

**DOI:** 10.1016/j.eclinm.2022.101568

**Published:** 2022-07-18

**Authors:** Emma Farrell, Eva Hollmann, Carel Le Roux, Joe Nadglowski, Deirdre McGillicuddy

**Affiliations:** a School of Education, University College Dublin, Belfield, Dublin, Ireland; b Diabetes Complications Research Centre, University College Dublin, Belfield, Dublin, Ireland; c Obesity Action Coalition, Tampa Florida, FL 33614, United States

**Keywords:** Obesity, COVID-19, Patient experience, Lived experience

## Abstract

**Background:**

People living with obesity are at elevated risk of hospitalisation, serious illness and mortality due to COVID-19. Little is known about their experience of living with obesity during the pandemic and its associated stay-at-home orders. This study sought to understand the experiences of people living with obesity during the COVID-19 pandemic.

**Methods:**

A stratified sample of Irish adults (*n* = 15) living with obesity engaged in open, phenomenological, interviews and a participatory photovoice methodology to capture both verbal and visual accounts of their experiences during the COVID-19 pandemic. Interviews, conducted throughout 2021, were transcribed verbatim and analysed thematically.

**Findings:**

Two overarching themes were identified. A) The pandemic and associated stay-at-home orders had a positive impact on the health and well-being of some participants; a negative impact on others; and this impact changed over time as the pandemic progressed. B) People living with obesity reported feeling stigmatised and ‘othered’ by their ‘at risk’ categorisation. Public health messaging and public discourse relating to obesity resulted in some people feeling segregated and punished by society.

**Interpretation:**

Changes in lifestyle initiated by the pandemic's stay-at-home orders had a varied impact on the health behaviours and outcomes of people with obesity. This variance offers helpful insight into the psychosocial aspects of obesity. Furthermore, the ‘othering’ effect of public health messaging during the pandemic warrants caution in light of the already stigmatised nature of this disease.

**Funding:**

This study is part of the SOPHIA project which received funding from the Innovative Medicines Initiative 2 Joint Undertaking under grant agreement No 875534.

**Research in context**

**Evidence before this study**

Large-scale studies have highlighted the negative impact of COVID-19 and its associated stay-at-home orders on the health behaviours and outcomes of people living with obesity. Concerns too have been raised at the increased presence of weight stigma in the media, healthcare settings and public campaigns during the pandemic.

**Added value of this study**

To the best of our knowledge, this study is the first to provide insight into the experiences of Irish adults living with obesity during the COVID-19 pandemic. It employed a range of novel qualitative methodologies and followed participants through ten months of the COVID-19 pandemic - capturing changes in their experiences as the country went into, out of, and back into periods of ‘lockdown’.

**Implications of all the available evidence**

This study highlighted how the impact of COVID-19 and its stay-at-home orders changed over time for people living with obesity. Furthermore, it highlighted how many living with obesity felt stigmatised and ‘othered’ by public health and media representations of obesity during the pandemic.

Alt-text: Unlabelled box

## Introduction

Severe acute respiratory syndrome coronavirus 2 (SARS-CoV-2) and its resulting novel corona virus disease 2019 (COVID-19) has resulted in more than five million recorded deaths globally since it was first identified in late 2019.^1^ From the initial stages of the pandemic, obesity was identified as a critical risk factor for complications of severe COVID-19.^2^ For example, a systematic review and meta-analysis of comorbidities associated with COVID-19 revealed obesity to be the second most prevalent comorbidity affecting 25% of the 125,446 patients studied.^3^ The authors took care to note the considerable geographic variation in their findings, and to highlight that the prevalence of obesity among COVID-19 patients reached up to 50% in the US, Mexico and the UK. Furthermore, a study of more than 6.9 million general practice patients in England found a significant positive linear association between increasing body mass index (BMI) and admission to ICU (Intensive Care Unit) due to COVID-19, with significantly higher risk for every BMI increase.^4^ The authors highlighted that “the mechanisms underpinning the association between obesity and sever COVID-19 outcomes remain elusive” (p.356). Current theories point to the metabolic effects of obesity on respiratory function, metabolic dysfunction, enhanced inflammatory response and impaired response to infection as possible mechanisms.^5^^,^^6^ Moreover, obesity has been linked to social disadvantage, smoking, age and gender – factors closely associated with poorer outcomes for COVID-19 patients.^7^ Indeed, the interactions between social disadvantage, non-communicable diseases, and COVID-19 have led some to describe the COVID-19 pandemic as a “syndemic”.^8^

Beyond the impact of the SARS-CoV-2 virus itself, the negative impact of the pandemic and its resulting stay-at-home orders on people living with obesity has also been charted. Globally, as the incidence of COVID-19 cases began to rise, national governments responded with measures such as stay-at-home orders; school and workplace closures; cancellation of public events and gatherings; policies on the use of face coverings outside of the home; restrictions on international and internal movement; and testing and contract tracing policies.^9^ Research published in this journal highlighted the detrimental impact of the first COVID-19 lockdown on people living with obesity in the UK in terms of health-related behaviours, mental health and access to weight management services.^10^ A large sample survey of UK adults^11^ also recorded the adverse impact of stay-at-home-orders on healthy eating and physical activity amongst adults living with obesity. Furthermore, authors have pointed to the potential association between increases in weight stigma during the COVID-19 pandemic and worse health outcomes for people living with obesity.^12^, ^13^, ^14^ For example, a scoping review by de Macêdo and colleagues,^15^ highlighted the presence of weight stigma in the media, healthcare settings, interpersonal relationships and public campaigns during the COVID-19 pandemic. The authors called for further research on the impact of weight stigma during the COVID-19 pandemic on people living with obesity. Furthermore, research on the experiences of adults categorised as at high risk of severe illness from COVID-19 revealed that many noticed that other people changed their behaviour towards them during the pandemic.^16^ Although not directly focused on the experiences of people living with obesity, this study highlights how adults in the ‘at risk’ category felt stigmatised by people not categorised as high risk.

This study aimed to examine the impact of the COVID-19 pandemic, and it's associated stay-at-home orders, on Irish adults living with obesity.

## Methods

### Design

Two complementary qualitative approaches were adopted to explore participants’ experience of living with obesity during the COVID-19 pandemic. The first approach was hermeneutic phenomenological in design with an open-interview method applied to understand participants’ experiences of the pandemic and its associated stay-at-home orders or ‘lockdowns’. Phenomenology is, as Merleau-Ponty^17^ suggests, that kind of human science approach that attempts to capture life as it is lived and give reflective expression to it. The central aim of hermeneutic phenomenology is to understand the lived experiences of other people and how they make sense of, or ascribe meaning to, these experiences.^18^ The second approach was a participatory image-based approach, fostering what Luttrell terms ‘visual rights’^19^ and a process of ‘collaborative seeing’^20^ through the use of photography. This approach enhanced the hermeneutic phenomenological approach by augmenting participants’ first-person descriptions with a more active and collaborative engagement between participant and researcher.

### Sample and recruitment

A non-probability, convenience, sample of participants was drawn from two Irish hospital-based weight management and diabetes management clinics. Convenience sampling, in contrast to probability sampling, does not aim to generate a sample representative of the entire population but rather “implies that a researcher is choosing informants because those informants might have something to say about an experience”.^21^ In this case, the experience under study, and the main criterion for inclusion, was the experience of living with obesity during the COVID-19 pandemic. The sample itself was stratified in advance of contacting participants to capture the experiences of people with Type 1 diabetes and Type 2 diabetes as well as the experiences of people at various stages in their weight management journey (Table 1). This included people who were engaged solely in lifestyle interventions, who were receiving pharmacotherapy, a sample that had undergone bariatric surgery and those who were not receiving treatment for obesity specifically but were attending a hospital diabetes clinic and had a BMI greater than 30 kg/m^2^. Researchers then generated a random list of participants under these five headings and contacted each participant by telephone, inviting them to participate. The aim was to achieve a sample size of 15, a sample size that is considered suitable for studies of this nature that focus on depth rather than breadth.^22^^,^^23^ Nineteen participants were contacted in order to generate the target fifteen, with four of those contacted choosing not to participate. The self-selection nature of participation is standard for convenience sampling but results in a degree of self-selection bias that is inherent in non-probability sampling. Ethical approval was granted by UCD's Human Research Ethics Committee (HS-20-12-McGillicuddy) and participants were aware that they could withdraw their participation at any stage without consequence. Consent was obtained from participants both in written form, in response to the study information sheet, and verbally before each interview.

**Table 1:** Sample characteristics. Table describing the number and gender of participants as stratified according to Type 1 or Type 2 diabetes or the form of treatment in which they are engaging. These included those engaged solely in lifestyle interventions, those undergoing pharmacotherapy and those who have undergone bariatric surgery.

	Male	Female
Lifestyle intervention	1	2
Surgery	2	2
Pharmacotherapy intervention	1	2
Type 1 Diabetes	1	1
Type 2 Diabetes	2	1
Total	7	8

### Data generation

Data were generated over a ten-month period from February to November 2021 – during which time Ireland was subject to varying ‘levels’ of restrictions according to the National Framework for living with COVID-19.^24^ These levels varied from national stay-at-home orders with all but essential services closed, to limited socialising and travel during period when there was a lower rate of COVID-19 infections. This ensured participants’ experiences of varying levels of restriction, as well as their anticipation, and eventual receipt, of the COVID-19 vaccination, were reflected in the data. All data were generated remotely using an online video conferencing platform by researchers with expertise in researching lived experience (E.F., E.H., D.McG.), one of whom is also a practicing nurse (E.H.). Participants were interviewed at at least two time points over the ten-month period with some participants engaging in up to four interviews. By way of illustration, one participant engaged in a conversational interview in February 2021, two photovoice interviews in May 2021 and a final group audeincing session in November 2021. The timing of interviews was based solely on convenience. To expand on the example above, the participant was contacted in February and the conversational interview arranged at their convenience the following week. They then consented to participate in the photovoice methodology and the camera and information pack was posted to their home address. They were then offered time to take the photos as guided and then returned the disposable camera to the research team. By the time the disposable camera images had been developed and digitised, and a suitable time was selected by the participant, it was May. Photovoice interviews tended to take a greater amount of time and participants were offered the option of a second interview if the first went over one hour. This was the case for the illustrative participant and a second interview was arranged for the following week. This process of data collection continued throughout the summer and, when completed, all participants were contacted in the autumn and invited to come together for group audiencing sessions. The last of these was conducted in November 2021, a group that the illustrative participant happed to participate in.

The first methodology involved a series of in-depth one-to-one hermeneutic phenomenological interviews with 15 adults living with obesity. These interviews were open-ended, beginning with a single question: ‘can you tell me about your experience of living with obesity’. The interviewers (EF, EH) did not interrupt the participant except to clarify a point or detail (e.g. “that took place before you attended the clinic?”); describe what they had heard (“it sounds like that experience was extremely challenging”); or to invite participants to elaborate on the experience (“what was that like?”). Such an open approach offered participants the opportunity to share their experiences in their own words and in their own way without the curtailment of researcher-designed interview schedules or questions. A total of fifteen interviews were carried out, ranging in duration from 45 to 105 minutes. The average interview length was over one hour.

Following their phenomenological interview, participants were invited to participate in the second, photovoice, methodology. Thirteen of the fifteen participants chose to progress to this second stage and disposable cameras were posted to their home address along with guidance notes. This guidance asked them to take images that represented three areas – the first related to their fears associated with living with obesity; the second the hopes for obesity treatment and; the third related to their experience of living with obesity during the COVID-19 pandemic. Participants used the return envelope to send back their cameras for photo developing and an interview was arranged where participants discussed the nature and meaning of each photo with researchers (E.F., E.H. and D.McG.). These interviews lasted up to two hours with any interview extending beyond that time being rescheduled for a second session to avoid participant and researcher fatigue. Following each individual interview, participants identified five photos which best represented their experiences and were invited to discuss these at an online group ‘audiencing’ session. Five participants agreed to participate in two group audiencing sessions – four females and one male. Facilitated by the lead researcher, these group audiencing sessions offered participants the opportunity to share their experiences of living with obesity and enabled a deeper layer of discussion and understanding as the researcher took a ‘step back’ from the process and participants proactively shared and discussed their experiences together.

### Data analysis

This study focused on data relating to participants experiences of COVID-19 generated in the phenomenological interviews, photovoice interviews and photovoice group audiencing sessions. Following verbatim transcription, researchers removed all identifying information from the written data and participants were assigned a pseudonym to protect their anonymity. All data were initially analysed by two researchers (E.F., E.H.) to identify high-level themes and all data relating to the COVID-19 theme were analysed by an individual researcher (E.F.) using Braun and Clarke's^25^ six-step framework for thematic analysis. This framework involved (a) becoming familiar with the data by reading and rereading the data in their entirety; (b) generating initial codes by jotting down descriptive labels and key words; (c) taking these initial codes and sorting them into potential themes; (d) reviewing and refining these prospective themes in terms of how accurately they reflected the meanings in the data; (e) further refining and defining these themes and what they revealed about participants experience of COVID-19; and (f) formulating the findings as a ‘whole’ and presenting them as described below.

### Role of the funding source

The study's funding source had no role in study design, data collection, data analysis, data interpretation, or writing of the report. All authors had access to the dataset and supported the decision to submit for publication.

## Results

Two overarching themes were identified (a) the impact of COVID-19 on health and well-being and (b) the experience of being categorised as ‘at risk’.

### Theme 1: Impact of COVID-19 restrictions on health and well-being

COVID-19 and its associated restrictions or ‘lockdowns’ had a marked impact on the health and well-being of many participants in this study. For some, this impact was negative, charted by participants in terms of weight gain, reduced activity, boredom and the effects of loneliness and isolation. For others the change in lifestyle precipitated by Government restrictions offered more time to exercise, cook, spend with loved ones and connect with their creative interests and community. As this study spanned a total period of ten months, it offered the opportunity to capture changes over time as vaccines were introduced, new variants emerged, and the COVID-19 pandemic, and successive periods of restriction, wore on. Outlined below are three sub-themes associated with the impact of COVID-19 restrictions on health and well-being: negative impact, positive impact and varied impact reflecting how, for many, the experience was both positive and negative depending on the time, life or living circumstance.

#### Negative impact

For many participants COVID-19 restrictions were associated with weight gain:

“The experience of living with obesity during COVID-19?… Weight gain. And I know it's not just obese people, I think the majority of people gained weight” – Ada

“I I put up the COVID stone in the last year” [laughs] – Miriam

“When lockdown came, everything changed and then the weight it started going back on” – Cora

“The food intake went up, the number of take-aways went up, the amount of wine went up” – Peadar

A reduction in the availability of healthy food options was identified by several participants:

“We couldn't go out, we couldn't go anywhere, and I got fed up of cooking. Then there were all these [takeaway] menus in the house and so […] because we couldn't go out we had those” – Áine

For many, getting a takeaway represented one of the few available treats at a time, when, as Steve put it, “there's very little natural rewards in life at the moment”:

“I was kind of living from one treat to the next treat. My focus had gone off life and had gone on to when is the next treat? When am I having the next take-away, the next meal, the next bottle of wine?” – Peadar

For Ada, eating was related to boredom and “not having much to do”:

“I found that I was eating more sweets and comfort food during COVID, during the pandemic. So, yeah, I definitely, I have gained weight during that time. […] That's kind of pandemic thing, you don't have much to do so you probably eat a bit more” – Ada

For Keith his increased “fridge visits” were also related to boredom:

“In many ways it's boredom, you know, it's to get a break from the room and break from the computer […] whereas if you're in work, you just have a chat with somebody” – Keith

Working from home also had an impact on participant's activity levels. Many were affected by gym closures and the geographical limits associated with the earlier lockdowns while others who, as Rory put it, “sit down for a living”, found that they were increasingly sedentary:

“I love to walk the beach [but] it came in under the category of COVID restrictions [as] it's 7km away from the house. […] That was just one of the things I really missed” – Áine

“I found that hard I have to say, the gym being closed. […] The weight resistance was really good and it was working, and I was really enjoying it and feeling really good, and then the lockdown came, and I hadn't had my knee surgery, so walking wasn't really an option, you know? And I found that very difficult then” – Catherine

“That [picture] is the new office chair that I bought during lockdown, because I was confined to barracks, and I suppose the word that comes to mind is sedentary. I tended to live a very sedentary life.” – Peadar

While the quotes above point to the negative impact COVID-19 restrictions had on the health and well-being of many participants, for others they represented a more positive experience.

#### Positive impact

The closure of cafés, bars and restaurants meant that April started bringing a lunchbox to work in place of her daily visit to a local café. She also described how, in the absence of dining and social activities, she developed the habit of visiting her parents every weekend and cooking dinner:

“I was never a baker or a cook or anything like that but I'd make dinner for the four of us every Saturday […] I'd make a desert as well, I'd make it all from scratch. […] That was actually a nice memory from COVID because I never did that before and it was nice” – April

Keith described how he lost weight over the first six months of the pandemic when his usual long daily commute to work was replaced by working from home:

“I was using the time I would normally spend in a car out getting exercise. I got back cycling, I'm probably fitter than I have been for a few years.” – Keith

Time emerged as a key theme for many participants – time to reflect, to pursue creative interests, to connect with others and to spend with family and loved ones.

Angela described how the lockdowns “gave me time to reflect”:

“It [lockdown] gave me time to tease out what I wanted in life and, where I was going, not just the weight, but every other aspect of my life, […] Covid gave us time to ourselves and with our family and the important things started to show.” – Angela

Having time to herself and her family had a very positive impact on Angela's well-being. She says “there were huge positives” associated with the period of restrictions and “I wouldn't say I'm alone with the goodness of the pandemic, you know, that lockdown period was really good”.

Peadar too described how he had “a great lockdown”, particularly during the first ten-week lockdown in Spring 2020.

“We had a really lovely time. Okay, so we couldn't go anywhere, but the fact that we couldn't go anywhere was really great, and we kind of did things we'd never done before and we had a great old time at one level, you know?” – Peadar

Keith described how the lockdown has forced him to reflect on his pre-pandemic lifestyle:

“Living a life in a car to and from work or living a life to and from airports, I don't necessarily think it suits a lot of people. We just do it because we have to do it. If I had a choice, I certainly would, certainly now, prefer to work at home or closer to home. I have never been able to just walk into work. […] I think the world we have ended up with in terms of work, it's just not good for the human spirit” – Keith

“I have to say it's been a good pandemic” – Keith

#### Varied impact

Keith was one of the participants who distinguished between his experience of the first major lockdown (March – May 2020) and the second (October 2020 – April 2021). This variance was associated with variables such as the weather, work and home conditions, and fatigue associated long periods under Government ‘stay-at-home’ orders.

“The first lockdown was great because the weather was so beautiful. But the most recent one I think has been difficult. Certainly, I found it difficult.” – Keith

Ada was one participant whose job was impacted by the pandemic. She was made redundant during the first lockdown and described how she “definitely fell into a depressive hole”:

“I just felt I was going in a downward spiral with my mental health. [In the city] you're surrounded by people, but you just feel so lonely. And the whole pandemic for people living on their own, this is the worst you know?” – Ada

Ada made the decision to move out of the city and to rent a house from a friend in the countryside. This move, precipitated by the pandemic and its effects, had a positive impact on her health and well-being:

“I'm lucky I'm six weeks in this wonderful place, and I'm calling [it] my six weeks of physical and mental health” – Ada

Peadar too described the ways in which his experience of the first lockdown, when his grown-up children returned home to spend the period with their parents, was very different to his experience of the second:

“So, the first lockdown from March to whenever last summer, they [children] were all here and we had great support, great times, great barbeques, getting back to nature and all that kind of stuff. This time the lockdown at Christmas we were on our own, they all went back and decided not to come home every weekend because of the COVID they stayed in [city] for a lot of the weekends“ – Peadar

#### Theme 2: The experience of being categorised as ‘at risk’

From the early stages of the pandemic, the Irish Health Service Executive (HSE), in line with many other national health agencies, declared individuals with obesity as ‘high risk’ for complications of COVID-19. The government advised that those over the age of 70, and those in this high-risk group, to ‘’stay at home as much as possible and to limit physical contact with other people”^26^

Being identified as high risk was significant for those who participated in this study.

Steve described how realising he was in a high-risk category was “definitely a wake-up call”:

“It kind of scares me, you know? [I] realise that if I got COVID that I would probably end up being very sick, that's scary, you know?” – Steve

Angela too spoke about the “fear” of being “at-risk because of being overweight” – “its always hanging over you”:

“It's the language around COVID; obesity is named constantly as at-risk […]. I know I'd have a better chance of survival if the weight was off”

**Figure 1:**
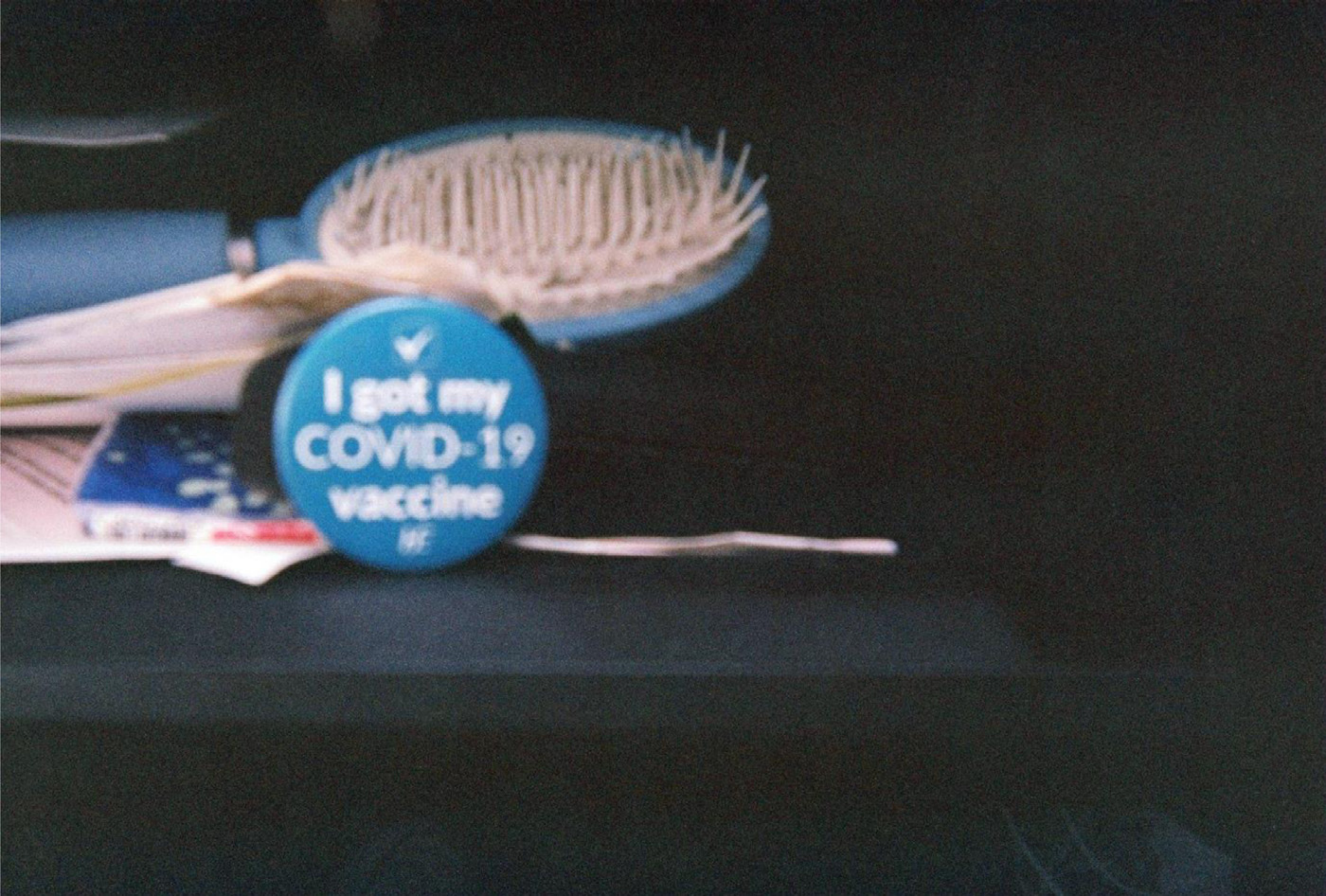
Photograph of Angela's (pseudonym) ‘I got my COVID-19 vaccine’ badge. Image generated as part of photovoice methodology resulted in insights into the segregative nature of the ‘at risk’ categorisation.

Sharing a photograph of her Health Service Executive “I got my COVID-19 vaccine” badge (Figure 1), Angela talked about segregation. She discussed the segregative nature of policies for those ‘at-risk’ and the sense this segregation was a punishment for being overweight. She compared this to the ‘punishment’ levied at those who are unvaccinated in terms of restricted access to social spaces and events.

“The policies at the moment are segregating, it's not your choice of isolation, it's systemic isolation. […] There is a ‘them and us’ sort of scenario with the segregation. It's not a nice place to be on either side of the fence in society. You're being punished for not being vaccinated but in many ways you're being punished for being overweight as well. Isolated from participation in society in subtle ways” – Angela

Freida also spoke about the damning narrative around obesity during the pandemic:

“At the beginning of COVID when people were talking about the obesity and you know, it just sounded as if it was all obese people that were blocking the beds in the hospital and basically they should be eradicated, you know? It was just horrible, I was afraid to nearly go out at all at that time” – Freida

Ada also spoke about how the public discourse and portrayal of people with obesity made her “feel like an outsider”:

“The conversation […] was, oh you know, ‘these are all overweight people and because of that they are high-risk’. There was even a comedy show on RTÉ (national broadcaster) and the comedian was on Grafton Street saying ‘Oh, fattie, don't come near me because you're high risk’” – Ada

“It's not nice when you're portrayed in that way in the media […] that makes you feel like an outsider, you know […]. It's almost like a zoo sometimes, how people look at you, and you're just ugly. And now on top of that you're also sick so you're high-risk. No, it doesn't make you feel good at all” – Ada

## Discussion

This study and its findings highlight two important points regarding the experiences of people living with obesity during the COVID-19 pandemic. The first is that this experience is not homogenous. Just as the pandemic experience of wider society is influenced by a range of variables such as work, living, personal and financial situation, so too is the experience of those living with obesity. While the experience may be a ‘mixed bag’, its contents warrant unpacking and examination as these social, personal, and environmental factors offer helpful insights into what can have a positive and negative impact on the health and well-being of people living with obesity. Participants in this study pointed to the poor availability of healthy and affordable take-away food options as well as the closure of gyms as having a negative impact on their health. This, combined with the curtailment of exercise to within 5km of home, made exercise and activity a challenge for many participants. Working from home and limited travel compounded this reduced activity and a number of participants “tended to live a very sedentary life” as Peadar described it. These findings support larger, quantitative, studies that highlight the negative impact of the pandemic on health-related behaviours.^10^, ^11^, ^12^ Furthermore, these issues point to the importance of making ‘the healthier choice the easier choice’,^27^ and how, in the midst of pandemic restrictions, making healthier choices presented a particular challenge for people living with obesity.

The change in lifestyle and working conditions precipitated by the pandemic and national measures, also presented an opportunity for many. Working from home meant that participants no longer had to spend long hours commuting to and from the office which, in Keith's case, meant that he could use “the time I would normally spend in a car out getting exercise”. In the wake of pandemic-related redundancy, Ada had the opportunity to move to the countryside which had a positive impact on her health and well-being while April described how she appreciated the time to cook meals for her family “from scratch”. Time emerged as a key theme for participants – time to reflect on what is important in life, time to spend with family and completing creative pursuits. Participants almost apologised for admitting “we had a really lovely time” (Peadar) but the changes brought about by restrictions afforded opportunity to re-evaluate life and working conditions and connect more with others.

The longitudinal nature of this study, albeit just ten months, allowed for reflection on the changing nature of experiences during COVID-19. Just as the experiences within the sample were not homogenous, the experiences at an individual level changed over time. These changes were shaped by factors such as the weather; change in living circumstances; and the wane in energy from the “in this together”^28^ of the first lockdown to a longer-term reality of “living with COVID-19”^24^ associated with the second. These points offer a reminder of the manner in which seemingly subtle changes can shape an alter the nature of patient experience. While research on the experience of people with obesity during the pandemic has identified risks in terms of complications and outcomes^29^^,^^30^ as well as treatment and care needs,^14^ the phenomenological and participatory nature of this study offered insight into the issues that were to the fore for participants themselves rather than their views on subjects identified by the research team. These data point to the social factors that are essential to effectively addressing obesity and other health inequalities during the COVID-19 pandemic.^31^

The second major point of discussion relates to participants experience of being identified as ‘at-risk’ of complications due to COVID-19. They described the fear and sense of segregation that resulted from this categorisation. Several participants felt “like an outsider” (Ada) amongst the general population – particularly as a result of the portrayal of people with obesity in the media: “it just sounded as if it was all obese people that were blocking the beds in the hospital and basically, they should be eradicated” (Freida). Extensive research points to the manner in which people with obesity are stigmatised by the media,^32^^,^^33^ healthcare providers^34^ and broader society.^35^, ^36^, ^37^ Flint^33^ raised concerns about the stigmatising media portrayal of obesity during the early stages of the COVID-19 pandemic, pointing to “a steady undertone of stigma toward people living with obesity” in the UK media. This study highlights how this undertone, or more overtly stigmatising comments such as that above, impacts people living with obesity.

While being identified as ‘at-risk’ meant that participants could take critical extra measures and were prioritised for vaccination, it also had an impact on their identity and sense of belonging: “you're being punished for being overweight […]. Isolated from participation in society in subtle ways” (Angela). Little is known about the impact of the ‘at-risk’ categorisation on those living with obesity and this study points to the potentially ‘othering’ effect of this categorisation. Othering, according to Powell and Menendian,^38^ is a process based on the conscious or unconscious assumption that a certain identified group poses a threat to a favoured group. In this case it is the conscious or unconscious assumption that people with obesity, by virtue of their vulnerability to the effects of COVID-19, pose a risk to the ‘healthy’ population by, for example, “blocking the beds in the hospital” as Freda put it. This finding points to the importance of service user involvement in the development and dissemination of public health advice so as to ensure that messages are both accurate and appropriate. This would help circumvent the need for clarification, sure as Ireland's Health Service Executive's clarification that people with obesity were not more likely to contract or transmit COVID-19 but rather more likely to experience severe complications associated with the virus, and ensure that public health messaging does not inadvertently ‘other’ or stigmatise the population it is intended to serve.

In conclusion, the COVID-19 pandemic has had wide ranging impacts on people living with obesity. Many of these were negative, including weight gain and isolation, while others were positive with time for reflection and health gain. The categorisation of, and narrative surrounding, people with obesity as “at-risk” of COVID-19 complications has resulted in many feeling stigmatised, segregated and ‘othered’. Understanding the legacy of this stigmatisation and the impact of COVID-19 restrictions on the health outcomes of people living with obesity is essential in providing an effective response to the disease as we move from pandemic to endemic.

## Contributors

Conceptualisation: D.McG., E.F.

Data curation: E.F., E.H., D.McG.

Formal analysis: E.F., E.H.

Funding acquisition: Cl.R., J.N., D.McG.

Investigation: E.F., E.H., D.McG.

Methodology: D.McG., E.F.

Project administration: D.McG., J.N., E.F.

Resources: Cl.R.

Software: NA

Supervision: D.McG., Cl.R.

Validation: All

Visualisation: E.F.

Writing – original draft: E.F.

Writing – review & editing: All

Approved final manuscript: All authors had full access to all the data in the study and had final responsibility for the decision to submit for publication**.**

## Data sharing statement

The data associated with this study will be made available in anonymised format from the corresponding author upon reasonable request.

## Declaration of interests

Cl.R. reports grants from the Irish Research Council, Science Foundation Ireland, Anabio, and the Health Research Board. He serves on advisory boards of Novo Nordisk, Herbalife, GI Dynamics, Eli Lilly, Johnson & Johnson, Glia, and Boehringer Ingelheim. Cl.R. is a member of the Irish Society for Nutrition and Metabolism. He was the chief medical officer and director of the Medical Device Division of Keyron in 2011. Both of these were unremunerated positions. Cl.R. was a previous investor in Keyron and was gifted stock holdings in September 2021 and divested all stock holdings in Keyron in September, 2021. He continues to provide scientific advice to Keyron for no remuneration. J.N. is the President/CEO of the Obesity Action Coalition and serves as volunteer co-chair of the Obesity Care Advocacy Network. E.F., E.H. and D.McG. have no conflicts of interest to declare.
